# Supplementary material for: Efficacy and Safety of Bevacizumab Plus Erlotinib in Patients with Renal Medullary Carcinoma
Source: Cancers (Basel). 2021 Apr 30;13(9):2170. doi: 10.3390/cancers13092170 (PMC8124338; doi:10.3390/cancers13092170)
Supplement: Supplementary file 1 [file cancers-13-02170-s001.zip › cancers-1144760-supplementary.pdf]

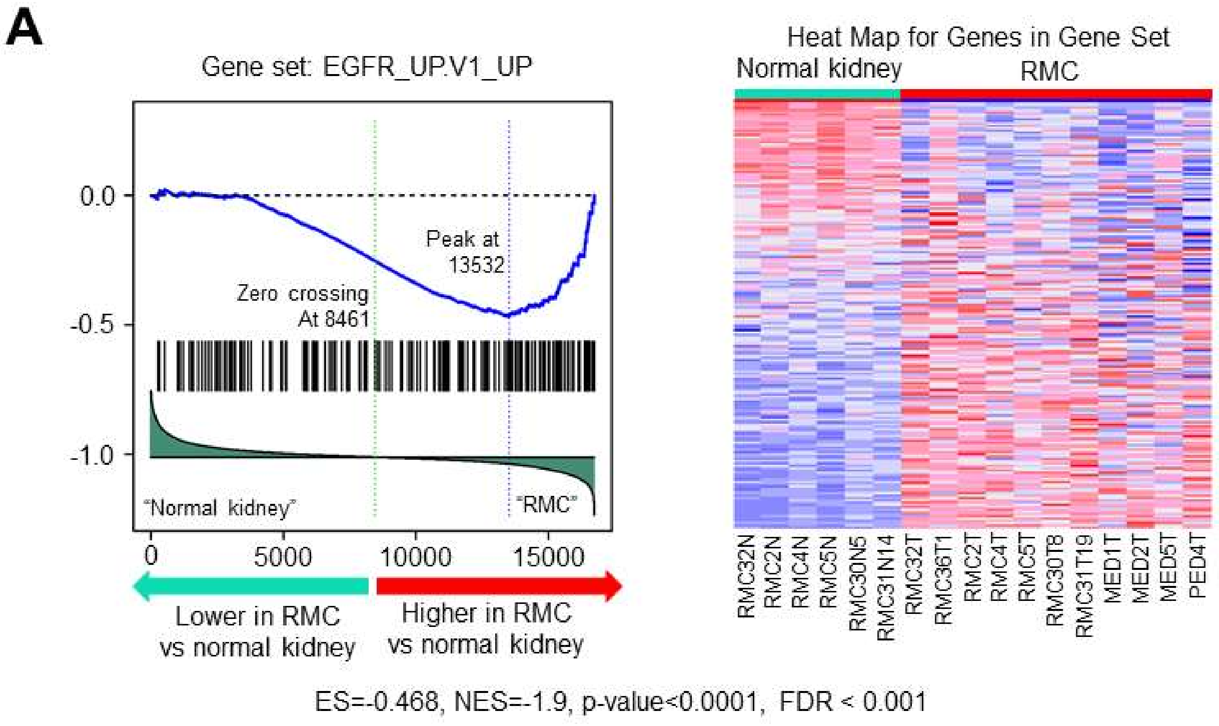


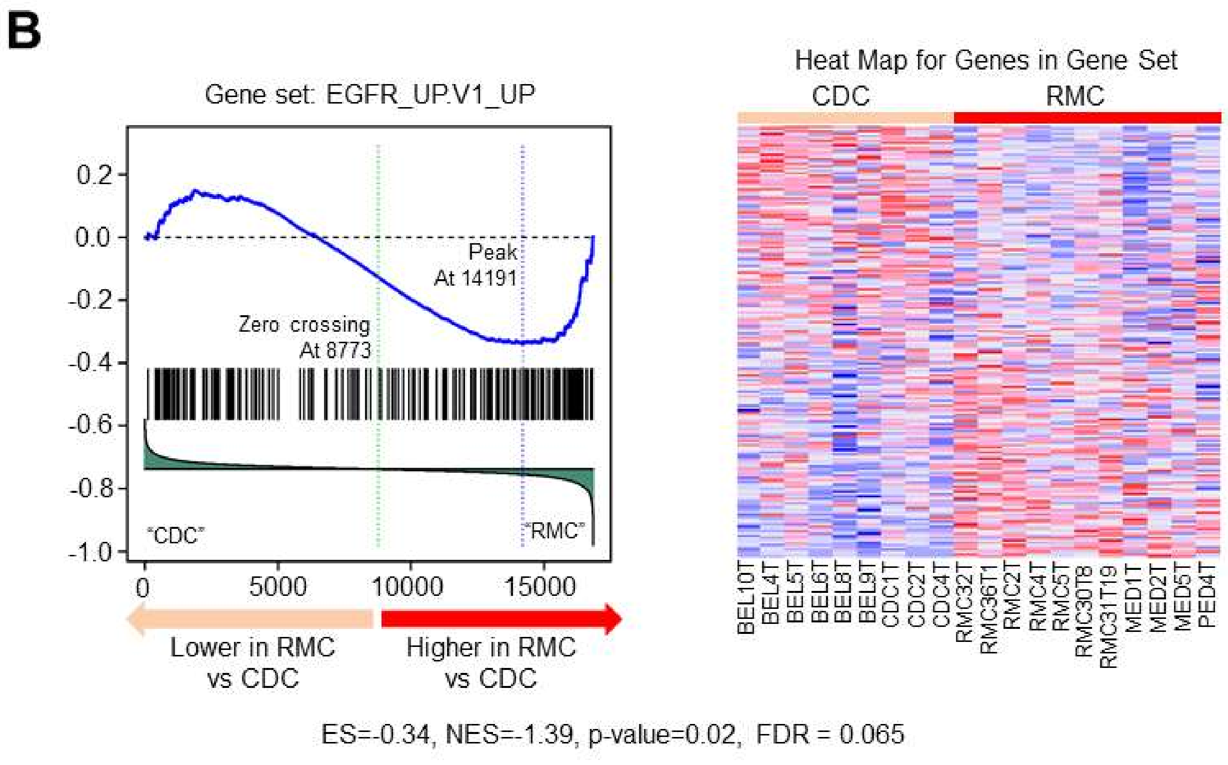


**Figure S1:** Gene set enrichment analysis (GSEA) of transcriptomic data of renal medullary carcinoma (RMC) tissues. This revealed a significant enrichment for the EGFR pathway in RMC compared with **(A)** adjacent normal kidney tissues or **(B)** collecting duct carcinoma (CDC). ES, enrichment score; NES, normalized enrichment score; FDR, false discovery rate. Data obtained from Msaouel et al. Cancer Cell (2020) 37: 1-15.

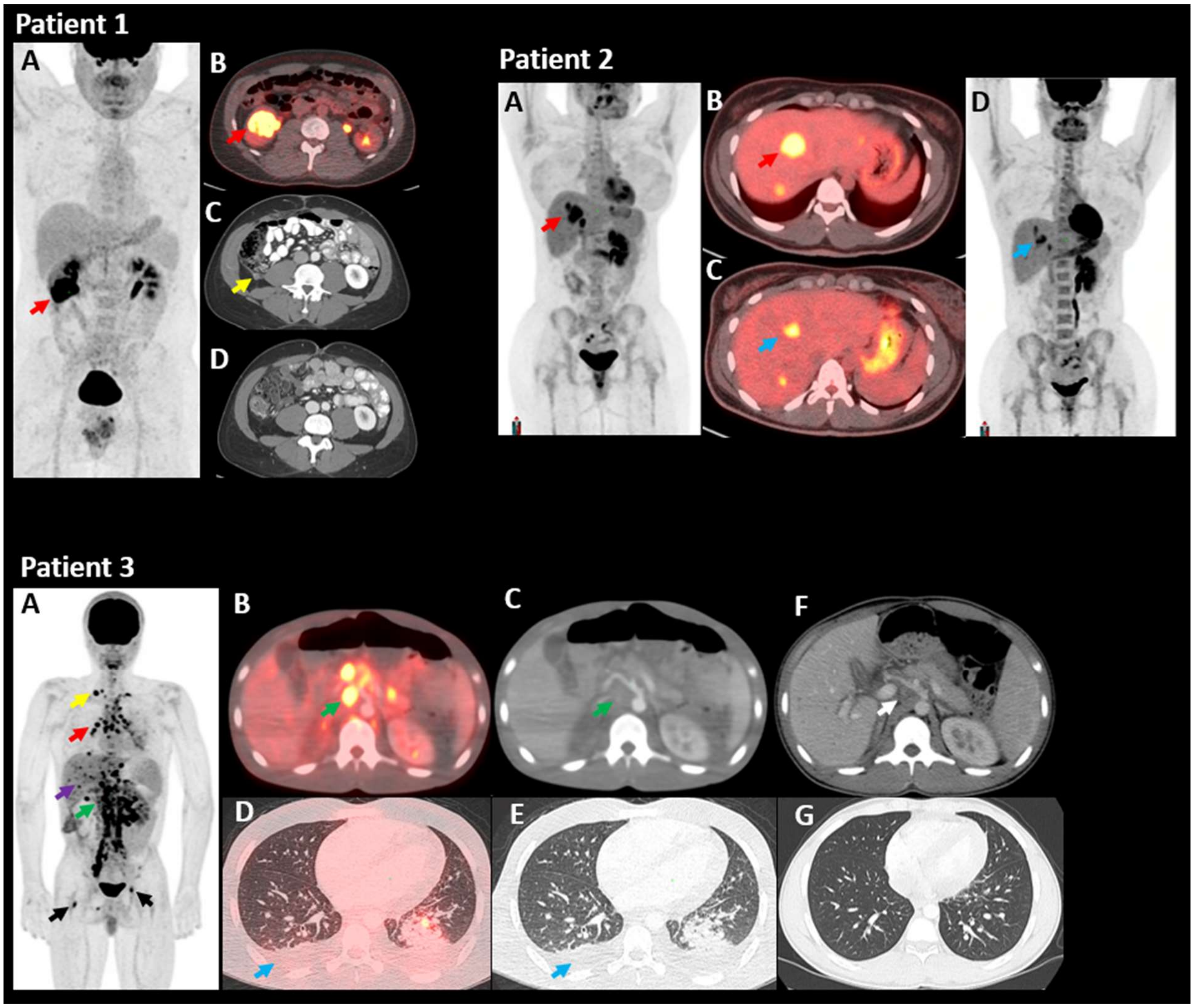


**Figure S2.** PET/CT of three different patients with renal medullary carcinoma (RMC) showing 18F-FDG avidity at different points during their treatment course. **Patient 1** was initially diagnosed with localized RMC in the inferior pole of the right kidney. 18F-FDG PET/CT (A) MIP (Maximum Intensity Projection) and (B) Axial fused PET/CT images demonstrating localized disease in the inferior pole of the right kidney (red arrows). The patient subsequently developed recurrent disease in the right retroperitoneal region seen on (C) Axial CT image (yellow arrow) with complete response on a follow up study following cytotoxic chemotherapy as seen on (D) Axial CT. **Patient 2** underwent right radical nephrectomy and subsequently developed metastatic RMC in the liver and thoracic spine treated with cytotoxic chemotherapy. 18F-FDG PET/CT (A) MIP (Maximum Intensity Projection) and (B) Axial fused PET/CT images demonstrating FDG avid hepatic metastases (red arrows) with treatment response on a subsequent 18F-FDG PET/CT scan as seen on (C) Axial fused PET/CT images and (D) MIP (blue arrows). **Patient 3** underwent a right radical nephrectomy for localized RMC and subsequently developed metastatic disease in the cervical, thoracic, and abdominal lymph nodes, liver, mesentery, and lumbar spine treated with cytotoxic chemotherapy. 18F-FDG PET/CT (A) MIP (Maximum Intensity Projection) demonstrates FDG avid cervical (yellow arrow), thoracic (red arrow), abdominal nodal metastases (green arrow), hepatic metastasis (purple arrow) and osseous metastases (black arrows) (B,C) Axial fused PET/CT and CT images demonstrate bulky FDG avid upper abdominal lymphadenopathy. (D, E) Axial fused PET/CT and CT images demonstrate bilateral malignant pleural effusions (blue arrows) and left lower lobe metastasis. Follow up (F, G) Axial CT images demonstrate treatment response with decrease in size of the upper abdominal nodal disease (white arrow) and resolution of the pulmonary findings.

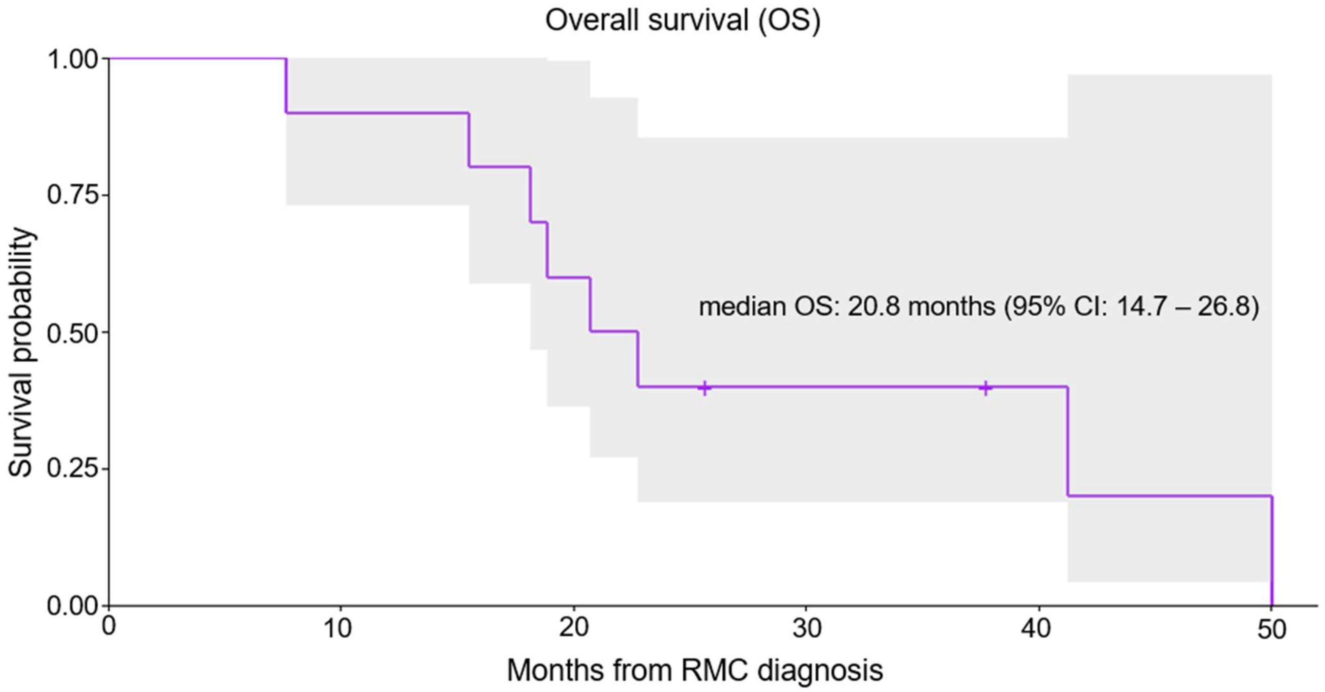


**Figure S3:** Overall survival from renal medullary carcinoma (RMC) diagnosis. The shaded areas represent the 95% confidence bands. CI, Confidence interval.
